# Prenatally Diagnosed Cardiac Tumors and Tuberous Sclerosis Complex: A Single-Center Experience

**DOI:** 10.3390/children12010094

**Published:** 2025-01-16

**Authors:** Matija Bakoš, Dora Jelinek, Ana Ćorić Ljoka, Nada Sindičić Dessardo, Dalibor Šarić, Ruža Grizelj

**Affiliations:** 1 Department of Pediatrics, Division of Pediatric Cardiology, University Hospital Centre Zagreb, 10000 Zagreb, Croatia; matija.bakos@kbc-zagreb.hr (M.B.); saric.dalibor@gmail.com (D.Š.); 2 Department of Pediatrics, Division of Neonatology and Neonatal Intensive Medicine, University Hospital Centre Zagreb, 10000 Zagreb, Croatia; dora.jelinek@kbc-zagreb.hr (D.J.); ana.coric.ljoka@kbc-zagreb.hr (A.Ć.L.); nada.sindicic.dessardo@kbc-zagreb.hr (N.S.D.); 3 School of Medicine, University of Zagreb, 10000 Zagreb, Croatia

**Keywords:** cardiac rhabdomyoma, cardiac tumors, prenatal diagnosis, tuberous sclerosis complex, mTOR inhibitors, pancreatic neuroendocrine tumor, fetus, neonate

## Abstract

Background/Objectives: Cardiac rhabdomyoma (CR), the most frequently occurring fetal cardiac tumor, is often an early marker of tuberous sclerosis complex (TSC). This study evaluates outcomes of fetuses with prenatally diagnosed cardiac tumors managed at a single tertiary center. Methods: Medical records of fetuses diagnosed with cardiac tumors between 2009 and 2024 were retrospectively reviewed. Results: Sixteen cases were identified, with a median follow-up of 6.7 years. TSC was confirmed in 14 cases (88%). Multiple tumors were observed in 13 cases (81%), while 3 cases (19%) had solitary tumors. Both non-TSC cases involved solitary tumors. Cardiac complications (arrhythmias, conduction disorders, and hemodynamic abnormalities) occurred in 38% of cases prenatally and 69% postnatally, with larger tumor diameters significantly associated with complications (*p* = 0.02). No fetal hydrops or mortality occurred; however, one child died at age five due to a seizure. Postnatal tumor regression occurred in 56% of cases and complete regression in 38% by a median age of 2.3 years (range: 0.6–4.4). One tumor remained stable. Brain MRI revealed TSC-related changes in all TSC-affected patients except one, who had a developmental brain anomaly. Most TSC patients experienced epilepsy (71%) and developmental delays. Conclusion: While CRs are typically benign and regress spontaneously, their strong association with TSC highlights the importance of early diagnosis and family counseling. TSC-related epilepsy and psychomotor delays significantly impair the quality of life. Early mTOR inhibitor therapy offers promise in mitigating TSC-related complications and improving outcomes.

## 1. Introduction

Primary cardiac tumors in children are extremely rare, with an incidence of approximately 0.2% [1]. Among these, cardiac rhabdomyomas (CRs) are the most common, accounting for over 60% of primary cardiac tumors during fetal life and childhood [2,3]. CR incidence ranges from 0.02% to 0.17% in live births, with a prenatal incidence of 0.12% [4]. Typically detected between 20 and 30 weeks of gestation, CRs are most often located in the ventricles and interventricular septum, with tumor sizes varying from a few millimeters to several centimeters. While usually benign and regressing spontaneously within the first years of life, their size, location, or growth can cause significant complications, including hemodynamic disturbances and life-threatening arrhythmias, both prenatally and postnatally.

CRs are also a hallmark of tuberous sclerosis complex (TSC), a rare multisystem genetic disorder caused by mutations in the *TSC1* or *TSC2* genes [5]. Prenatal detection of CRs carries a 75–80% risk of TSC, particularly when multiple tumors are present [6,7,8]. Early diagnosis, comprehensive surveillance, and timely management are critical to mitigating the severe morbidity and mortality associated with TSC. A definitive diagnosis of TSC is typically achieved through genetic testing, though up to 10–25% of patients may lack detectable mutation using conventional genetic methods [9]. In such cases, diagnosis relies on established clinical criteria [9].

By targeting the underlying pathogenic mechanism of TSC, mTOR inhibitors (mTORis) have become a cornerstone in the treatment of TSC-associated tumors across various organ systems [9]. Emerging evidence demonstrates that mTORis effectively reduces CR size and alleviates associated symptoms [10]. Their use has also been successfully documented in severe prenatal cases [11]. However, due to their potential risks and the tendency of CRs to regress spontaneously, mTORis are reserved for life-threatening situations, such as severe hemodynamic compromise or malignant arrhythmias, to avoid high-risk invasive procedures [10]. Beyond cardiac benefits, mTORis addresses systemic manifestations of TSC, including subependymal giant cell astrocytomas (SEGA) and early-onset epilepsy, improving long-term neurological and cognitive outcomes [12].

This study presents a retrospective case series of neonates with prenatally diagnosed cardiac tumors managed at a single tertiary center. It aims to evaluate the prenatal and postnatal features and outcomes of CRs and to assess the broader impact of TSC on long-term health. By elucidating the natural course of CRs and associated TSC manifestations, this study emphasizes the significance of early diagnosis, family counseling, multidisciplinary management, and the potential of emerging therapies like mTORis in improving outcomes for affected neonates.

## 2. Material and Methods

This retrospective case series reviewed fetal echocardiographic (ECHO) screenings performed between January 2009 and January 2024 at the Department of Pediatrics, University Hospital Center, Zagreb, Croatia. This study included fetuses diagnosed with cardiac tumors, with data obtained from the hospital’s digital database and medical records. The information encompassed prenatal, perinatal, and postnatal findings.

### 2.1. Settings and Prenatal Diagnostic Workflow

The University Hospital Center, Zagreb, is the largest Croatian tertiary referral center for prenatally detected fetal anomalies requiring advanced diagnostic evaluation. It does not have a maternity ward. When a cardiac tumor was detected or suspected on fetal ultrasound, expectant mothers were referred to the Outpatient Clinic for Fetal Echocardiography at our institution for detailed evaluation. Two certified pediatric cardiology specialists with extensive expertise performed transabdominal fetal ECHO using a high-resolution ultrasound machine (GE Vivid E9, GE Vivid E90). Our study adhered to the International Society of Ultrasound in Obstetrics and Gynecology (ISUG) guidelines for fetal cardiac screening, ensuring a thorough and standardized assessment of the fetal heart [13]. The prenatal ECHO protocol included standard measurements of cardiac structures, including ventricular, atrial, and outflow tract dimensions, and the evaluation of potential structural abnormalities. We meticulously documented the number, size, and location of any cardiac tumors observed during this examination. Doppler flow analysis evaluated blood flow patterns across heart valves and major vessels, offering valuable insights into the fetus’s hemodynamic status. Hemodynamic disturbances and conduction irregularities, including arrhythmias, were assessed to provide a comprehensive evaluation of fetal cardiac function. Follow-up ECHO evaluations were scheduled at intervals of two to four weeks to monitor tumor progression and detect emerging complications, with more frequent assessments conducted if clinically indicated. After birth, all neonates underwent a thorough diagnostic workup, including brain MRI, abdominal ultrasonography, dermatologic and ophthalmologic examinations, seizure assessment, and genetic testing.

### 2.2. Data Collection

Demographic data included maternal age, parity, family history of TSC, gestational age (GA) at diagnosis, mode of delivery, GA at birth, birth weight, and Apgar scores. Prenatal findings focused on ECHO parameters such as tumor size, number, and location, as well as the presence of arrhythmias and overall fetal hemodynamic status. Postnatal evaluations included ECHO, electrocardiograms, neurologic assessments, electroencephalograms, brain magnetic resonance imaging (MRI), dermatological and ophthalmological examination, abdominal ultrasonography, and genetic testing. TSC diagnosis was confirmed using the criteria established by the International Tuberous Sclerosis Complex Consensus Group [9].

### 2.3. Statistical Analysis

Descriptive summaries of demographic data, cardiac tumor characteristics, associated clinical features, and outcomes are provided. Continuous variables (e.g., GA, birth weight) were reported as means with standard deviation [SD] or medians with ranges. Categorical variables were presented as counts and percentages. Comparison of continuous variables was conducted using the Student *t*-test, with *p* < 0.05 considered statistically significant.

The study protocol was approved by the Ethics Committee of University Hospital Center, Zagreb.

## 3. Results

During the study period, 15 pregnancies involving 16 fetuses with prenatally diagnosed cardiac tumors were identified. The cohort included 13 singleton pregnancies and 2 dichorionic twin pregnancies. In one twin pregnancy, one fetus was unaffected by CR and other features of TSC, while the other twin, along with both fetuses in the second twin pregnancy, were affected by CRs and TSC. The median maternal age was 30.5 years (range: 23–38), with one-third of mothers being primiparous. None of the pregnancies were terminated.

A positive family history of TSC was noted in two pregnancies. In the first case (patient No. 2, Table 1), the mother had a pre-existing diagnosis of TSC (with a history of nephrectomy due to angiolipoma), as did her older son and maternal sister. In the second case, involving twins (patients No. 9 and No. 10, Table 1), TSC was present on the paternal side, with multiple family members affected, including the father, his sister, mother, and grandmother. In the remaining 11 cases, family history was negative, and detailed clinical assessment of family members did not reveal any features of TSC. However, as not all parents underwent genetic testing, the possibility of inheritance from an undiagnosed parent with subtle features of TSC cannot be excluded. Notably, the proportion of de novo mutations in our cohort (85%) is higher than the expected incidence of 70%.

Among the 16 live-born cases, 8 (50%) were female. The median GA at birth was 39 weeks (range: 36–41), and the median birth weight was 2.940 g (range: 2.190–4.330). Half of the deliveries were performed by the cesarean section. In addition to standard obstetric indications, two planned cesarean sections were conducted due to severe hemodynamic complications in two fetuses and one due to fetal bradycardia. All neonates were delivered at term (*n* = 12) or near-term (*n* = 4).

Among the 16 neonates, 14 (88%) were diagnosed with TSC; 7 were confirmed by *TSC1/2* gene mutations, and 7 were based on clinical diagnostic criteria [9]. Two neonates had isolated cardiac tumors without clinical or genetic evidence of TSC. The median follow-up period was 6.7 years (range: 1–15.3). Detailed clinical information is provided in Table 1.

### 3.1. Tumor Characteristics

**Figure 1:**
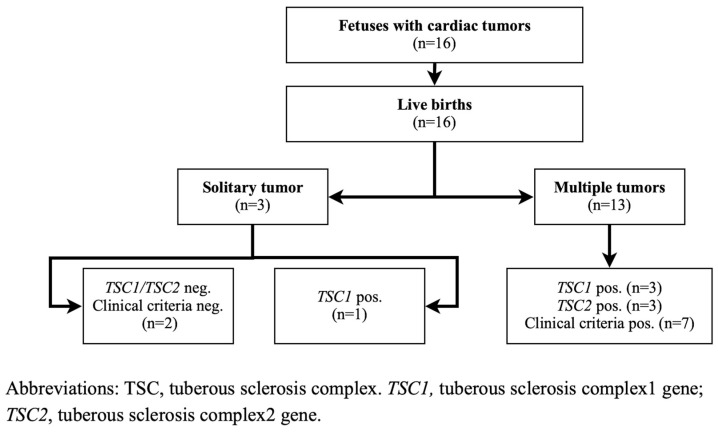
Flow diagram of neonates prenatally diagnosed with cardiac tumors.

The median GA at cardiac tumor detection was 31 weeks (range: 21–37). Multiple tumors were observed in 13 patients (81%), while 3 patients (19%) had solitary tumors. Tumors predominantly involved the ventricles, with the left ventricle (LV) being the most common site (81%), followed by the right ventricle (RV) (69%) and interventricular septum (IVS) (63%). Atria involvement was observed in three patients (19%). Tumor sizes ranged from a few millimeters to a maximum of 25 × 30 mm. Both patients without TSC had solitary tumors, whereas nearly all TSC patients, except one, presented with multiple tumors (Figure 1). Postnatal tumor regression was observed in nine patients (56%), with complete regression in six patients (38%) by a median age of 2.3 years (range: 0.6–4.4 years). Tumor size remained stable in one patient.

### 3.2. Cardiac Complications

Several patients experienced multiple cardiac complications, which included arrhythmias, conduction disorders, and hemodynamic abnormalities. Prenatally, 6 fetuses (38%) had a total of 10 complications, while postnatally, 11 neonates (69%) experienced 25 complications (Table 2).

Prenatal complications included significant arrhythmias in one fetus, hemodynamic disturbances (tricuspid regurgitation, right ventricular outflow tract turbulence, and left ventricular outflow tract [LVOT] obstruction) in three fetuses, and a combination of these with severe clinical consequences in two fetuses. There were no cases of hydrops fetalis or perinatal mortality.

All three patients with significant prenatal arrhythmias (patients No. 8, 11, and 14; Table 1) required prenatal and/or immediate postnatal treatment with corticosteroids, sotalol/lisinopril, and sirolimus/propranolol, respectively, leading to excellent clinical responses. Prenatal LVOT obstruction was resolved in all three cases, including one case (patient No. 16, Table 1) where the obstruction resolved spontaneously during the prenatal period. In another case (patient No. 14, Table 1), significant prenatal arrhythmias and progressive subaortic stenosis (80% obstruction, 40 mmHg gradient) were managed with early postnatal beta-blocker and sirolimus therapy. This resulted in complete regression of the largest CR (13 mm × 13 mm) in the LV and multiple CRs in the RV, as well as substantial regression of an IVS tumor causing LVOT obstruction, all by two months of age following the initiation of therapy.

Postnatally, three previously undetected patients presented with isolated conduction disorders, including severe repolarization disturbances, right bundle branch block, first-degree atrioventricular block with RV hypertrophy, and two patients with arrhythmias (ventricular extrasystoles, symptomatic ventricular and supraventricular tachycardias).

The group with cardiac complications had significantly larger maximum CR diameters compared to those without complications (*t*-test, *p* = 0.02).

### 3.3. Cerebral Manifestations of TSC

Postnatal brain MRI revealed TSC-related changes in 13 of the 14 TSC-affected patients (93%), with one patient presenting with a developmental brain anomaly. Subcortical tubers were observed in 10 patients (71%); subependymal nodules were observed in 11 patients (79%), and cerebral white matter radial migration lines were observed in 3 patients (21%). SEGA was diagnosed in four patients (Table 3). One patient (patient No. 6, Table 1) developed hydrocephalus due to a progressively growing SEGA (4 cm), requiring ventriculoperitoneal drainage and subsequent SEGA removal despite treatment with mTORi.

Epilepsy was observed in 10 patients (71%), with most being treated with a combination of antiepileptic drugs. Six of these patients achieved seizure control. Seven patients received mTORi therapy, resulting in seizure resolution in four patients and improvement in two. Psychomotor and developmental delays were prevalent in the majority of these children (Table 3).

### 3.4. TSC Comorbidities

Cutaneous manifestations were observed in 11 patients (79%), while renal abnormalities were identified in 7 patients (50%), including one case that required nephrectomy due to the rapid growth of renal angiolipoma. Ophthalmologic features were present in five patients (36%).

### 3.5. Neuroendocrine Tumors

At the age of 10, patient No. 7 underwent surgical resection of a nonfunctional pancreatic neuroendocrine tumor (PNET), graded as WHO Grade I. The tumor measuring 5.2 cm × 7 cm × 7.3 cm was incidentally identified during routine MRI surveillance for renal angiolipomas as part of TSC management.

## 4. Discussion

This study highlights the prenatal and postnatal characteristics of cardiac tumors, their strong association with TSC, and the clinical outcomes in affected neonates. In our cohort, 88% (14/16) of patients were diagnosed with TSC, with diagnoses confirmed in seven patients through postnatal genetic testing and in seven patients based on clinical criteria.

In our study, the earliest detection of a cardiac tumor occurred at 21 weeks of gestation, with a median diagnosis at 31 weeks. No cases of fetal hydrops or perinatal demise were observed, and all neonates were delivered at term or near-term. One patient died at the age of five following a seizure attack at home. Reported perinatal mortality rates in other studies range from 0% to 39%, with nearly all cases of fetal demise preceded by severe fetal hydrops, large tumor size, and fetal arrhythmia [14,15,16,17,18,19,20]. Variability in these rates may be attributed to “hidden mortality,” which refers to the exclusion of terminations of pregnancy cases from mortality statistics.

The high incidence of cesarean sections in our cohort (50%) reflects the complexities of managing pregnancies involving fetuses with cardiac tumors. This rate aligns with previous studies reporting cesarean delivery rates ranging from 38.9% to 70% in comparable cases [20,21,22]. The preference for cesarean delivery often arises from the need to address potential complications, such as fetal distress or hemodynamic instability, as observed in our cohort. These findings highlight the importance of individualized delivery planning for pregnancies with prenatal cardiac tumor diagnoses to ensure both maternal and neonatal safety.

### 4.1. Cardiac Rhabdomyomas and Tuberous Sclerosis Complex

In prenatal life, multiple CRs are frequently the earliest indicator of TSC, observed in over 80% of cases [6,7,8,10,23]. While both single and multiple CRs are included as primary diagnostic criteria for TSC [9], the risk of TSC in cases of a single isolated CR remains uncertain. A recent retrospective study of 240 fetuses with suggestive antenatal findings reported a definite TSC diagnosis in 50% (41/82) of cases with a single CR and 80.3% (127/158) with multiple CRs [24]. Similarly, Tworetzky et al. [25] diagnosed TSC in 30% (7/23) of children with a single tumor and 95% (61/64) of those with multiple lesions. In our cohort, prenatal ECHO detected multiple CRs in 81% (13/16) of fetuses, while a solitary tumor was identified in 19% (3/16). TSC was confirmed in all cases with multiple CRs. Among the three cases with solitary tumors, only one was diagnosed with TSC. This tumor exhibited atypical CR characteristics, being solitary, located in the right atrium, and arising from the lower interatrial septum. Notably, the literature suggests that only 2% of CRs are located in the RA [26]. In the other two solitary cases, genetic testing and the absence of clinical signs excluded TSC, with one tumor later identified as a lipoma via MRI.

### 4.2. Cardiac Complications

Cardiac complications were significant in this cohort. Prenatal complications, including arrhythmias and hemodynamic disturbances, occurred in 38% of cases (*n* = 6), while postnatal complications were observed in 69% (*n* = 11). This discrepancy reflects the superior sensitivity of postnatal diagnostic tools in detecting arrhythmias and conduction disorders compared to prenatal tools. Postnatal evaluations, including continuous 24-h rhythm monitoring and detailed electrocardiography, are more effective than prenatal assessments, typically limited to brief ECHO examinations. Additionally, prenatal diagnoses of cardiac tumors prompt thorough postnatal evaluations, increasing the likelihood of identifying rhythm disturbances and asymptomatic conduction disorders. A study by Altmann et al. [20] also reported a higher incidence of postnatal arrhythmias (41%) compared to prenatal detection (7%). Common postnatal arrhythmias in our cohort included repolarization and depolarization disturbances (31%), Wolff–Parkinson–White syndrome (19%), ventricular extrasystoles (19%), and paroxysmal supraventricular tachycardias (19%) (Table 2).

The size of CRs in fetuses and neonates is a critical factor influencing the risk of arrhythmias and hemodynamic disturbances. Larger CRs, particularly those exceeding 30 mm in diameter, are strongly associated with a higher incidence of postnatal arrhythmias requiring treatment, including ventricular tachycardia and conduction abnormalities [27]. Larger tumors also contribute to hemodynamic complications, such as ventricular inflow or outflow obstruction, which can exacerbate heart failure and potentially lead to life-threatening conditions and death [10,19]. Conversely, smaller CRs are less likely to cause severe complications, underscoring the importance of tumor size as a predictive factor for clinical outcomes [23]. In our cohort, the group with cardiac complications exhibited significantly larger maximum CR diameters compared to those without complications (*t*-test, *p* = 0.02). Our observations align with Peng et al. [23], who reported that the mean maximum diameter of CRs was substantially larger in patients experiencing intracardiac complications (17.92 ± 8.43 mm) compared to those without complications (10.64 ± 4.37 mm).

Postnatally, CRs typically undergo spontaneous regression during the first years of life. In our study, postnatal regression occurred in 56% (*n* = 9) of cases, with complete regression in 38% (*n* = 6) by a median age of 2.3 years, supporting the hypothesis that these tumors are generally self-limiting. Although CRs are often generally benign, they can occasionally lead to significant obstruction, hemodynamic compromise, and heart failure, depending on their size and location in both prenatal and postnatal periods. In rare, life-threatening situations where medical therapy is insufficient, surgical intervention may be required [28].

mTORi therapy has proven to be an effective non-invasive treatment option in managing such cases [29]. Recent systematic analysis [11] and a literature review [30], which include nine studies reporting on 11 fetuses with TSC treated with prenatal mTORi, underscore the potential of this therapy to reduce tumor size and improve hemodynamic stability in fetuses, supporting its role as a valuable non-invasive treatment option for severe CR-related complications. Notably, in contrast to the group that did not receive prenatal mTORi, there were no reported cases of fetal demise, neonatal death, or the need for cardiac surgery in the prenatal mTORi-treated group. This reduction in tumor burden is particularly significant for mitigating severe prenatal complications, such as fetal hydrops, and addressing potentially life-threatening cardiac symptoms in prenatal life.

In our cohort, the proximity of CRs to the ventricular septum caused prenatal LVOT obstructions in three cases. In one case, the obstruction resolved spontaneously during the prenatal period, while in another, the obstruction regressed postnatally as the tumor spontaneously decreased in size. In one notable case, severe subaortic stenosis and arrhythmias resolved entirely within two months of initiating mTORi therapy. Our case supports the growing body of evidence demonstrating the therapeutic effects of mTORi in resolving obstructive CRs and associated arrhythmias [11,31].

### 4.3. Cerebral Manifestations of TSC

Intracranial manifestations of TSC are strongly associated with a high risk of neurological impairment [32]. Epilepsy, one of the most prevalent and debilitating features, affects over 80% of individuals with TSC [33]. Seizures, often refractory to treatment, can manifest as infantile spasms, focal seizures, and generalized tonic–clonic seizures. Early onset and frequent seizures are closely linked to poorer neurodevelopmental outcomes, including cognitive impairments, autism spectrum disorders, and developmental delays. In our cohort, all patients had TSC-associated brain lesions, and 71% (10/14) developed epilepsy, with seizure control achieved in seven cases. Among those without epilepsy, 75% (3/4) attained normal neurodevelopmental outcomes, compared to only 20% (2/10) of those with epilepsy, both of whom achieved seizure remission. Seven children received treatment with mTORis, with 57% (4/7) achieving seizure control. Long-term cognitive and behavioral challenges persist in the majority of patients, with autism spectrum disorder diagnosed in 20%.

Fetal brain MRI is a valuable tool for detecting brain lesions in the majority of fetuses with TSC, with optimal timing from 22 weeks of gestation onwards as detection improves with advancing GA [34,35]. Combined with CR detection on fetal ECHO, MRI findings can confirm TSC even in the absence of genetic testing, though the lack of intracerebral lesions does not exclude the diagnosis. In our study, prenatal brain MRI was performed in five fetuses at a median GA of 31.5 weeks, identifying TSC-related brain lesions in four cases and confirming the diagnosis prenatally. Notably, in one fetus with a negative prenatal MRI, cerebral abnormalities were detected postnatally. Prenatal diagnosis provides significant benefits, including early parental counseling and informed decision-making. Since prenatal lesion detection has been linked to neurodevelopmental outcomes and an increased risk of autism [34], early diagnosis enables the initiation of preventive strategies, such as antiepileptic therapy (e.g., vigabatrin) and mTORis [9,36]. While research suggests that early treatment before the onset of seizures may reduce the risk of drug-resistant epilepsy and improve long-term outcomes [34,36,37,38,39], the recently conducted PREVeNT clinical trial reported that preventive vigabatrin treatment neither improves neurocognitive outcomes nor delays the onset or reduces the incidence of focal seizures or drug-resistant epilepsy in children with TSC at 24 months [40]. Recent studies highlight the potential of prenatal mTORis in managing TSC by targeting the hyperactivation of the mTOR pathway, a hallmark of the disease [30]. This approach is hypothesized to reduce the in utero burden of brain lesions, potentially mitigating severe neurological complications associated with TSC [41]. However, further studies are required to thoroughly evaluate their impact on neurological outcomes and to determine the optimal dosing, timing, and long-term safety for both the fetus and the mother [30].

### 4.4. Renal Involvement in TSC

Renal involvement is a significant aspect of TSC, commonly presenting as angiolipomas (70–80%), cystic kidney disease (50%), and renal cell carcinoma (3–5%). These conditions often coexist and tend to become more apparent with age. In our cohort, renal abnormalities were observed in seven patients (50%), predominantly as angiolipomas (86%), with one patient (14%) presenting with multiple cysts. Notably, one patient underwent a nephrectomy at the age of four due to the rapid growth of an angiolipoma. It is important to note that normal renal function and imaging in early childhood do not preclude the later development of renal lesions, highlighting the importance of continuous monitoring of renal function and imaging from the time of diagnosis in all individuals with TSC. Targeted therapies, such as mTORis, have demonstrated promising efficacy in managing renal manifestations by reducing the size and growth of angiolipomas. These findings underscore the importance of early detection and timely intervention to preserve renal function and mitigate disease progression. Recent guidelines by Mekhali et al. [42] provide detailed recommendations for the diagnosis, monitoring, and treatment of renal involvement in TSC.

### 4.5. Miscellaneous

The case of our patient with PNET highlights the critical importance of routine and detailed surveillance in identifying and managing rare extrarenal complications linked with TSC. Following the introduction of the 2012 TSC surveillance guidelines, there has been a significant increase in documented cases of nonfunctional PNETs among TSC patients [43]. To date, over 60 cases have been documented, with an estimated prevalence of PNETs in TSC patients ranging from 4% to 9% [44,45], significantly higher than the 0.003% prevalence in the general population [46]. This striking disparity suggests a potential link between TSC and an increased susceptibility to NETs. The biological basis for this association remains an active area of research. TSC is defined by mutations in the *TSC1* and *TSC2* genes, which regulate the mTOR pathway. Dysregulation of this pathway has been implicated in tumorigenesis and may contribute to the development of PNETs. However, unlike angiolipomas or CRs, which are typical benign hamartomas associated with TSC, PNETs exhibit distinct histopathological features and clinical characteristics. Consequently, they are not currently included in the diagnostic criteria for TSC [9]. The higher frequency of PNETs among TSC patients underscores the importance of regular surveillance imaging, which allows for early detection and timely intervention before these tumors become symptomatic or invasive. Continued research is needed to clarify the natural history of PNETs in TSC, optimize management strategies, and evaluate the potential benefits of including PNET surveillance in TSC clinical guidelines.

### 4.6. Limitations

This study is constrained by its retrospective design, along with the inherent limitations associated with such an approach and a relatively small sample size, which may affect the generalizability of our findings. However, the extended follow-up period, with a median duration of 6.7 years, provides valuable insights into the long-term outcomes of affected patients, contributing meaningful data to this area of research.

## 5. Conclusions

While many CRs regress spontaneously, their potential to cause significant cardiac complications, along with their strong association with TSC, underscores the need for a comprehensive, multidisciplinary management approach. Early diagnosis and timely intervention, particularly the use of mTORi, are pivotal in mitigating both the cardiac and neurological sequelae of TSC. These findings highlight the importance of individualized therapeutic strategies to optimize outcomes for affected patients.

## Figures and Tables

**Table 1:** Clinical characteristics of 16 cases of prenatally diagnosed cardiac tumor(s).

No.	TSC	Diagnosis	Mutation	GA at Diagnosis	Tm Number	Tm Location	Max ⌀ (mm)	Fetal Arrhythmia/ Hemodynamics	Neonatal Arrhythmia/ Hemodynamics	Tm Growth Pattern	Brain MRI	Epilepsy	Other TSC Manifestation	NDI
1	No	Genetic	Negative	23	Single	LV-IVS	14	None	VES Repolarization abnormality	No change	Normal	No	None	None
2	Yes	Clinical		28	Multiple	LV, RV, IVS	8	None	None	Regression	Dandy-Walker sy Porencephaly	Yes	Skin lesions	Severe
3 ^a^	Yes	Genetic	*TSC1*	33	Multiple	LV, RV	10	None	None	Complete regression	SEGA	No	Skin lesions Retinal achromic patches	None
4	Yes	Clinical		34	Multiple	LV, RV, IVS	16	None	Repolarization abnormality	Regression	Cortical tubers SENs	Yes	Skin lesions Renal cysts Thoracic scoliosis	Severe Autism
5	Yes	Clinical		33	Multiple	LA, LV, RV, IVS	22	TR	MR	Regression	SENs	Yes	Skin lesions Renal angiolipomas	Moderate
6	Yes	Clinical		31	Multiple	RV, IVS	10	None	Right bundle branch block	Regression	Cortical tubers SENs SEGA	Yes	Skin lesions Retinal achromic patches Optic disc hamartoma	Severe
7	Yes	Genetic	*TSC1*	35	Multiple	LV, RV, IVS	20	None	1st-degree AV block RV hypertrophy	Regression	Cortical tubers SENs WM migrational abnormalities	Yes	Skin lesions Renal angiolipomas PNET	None
8	Yes	Genetic	*TSC1*	31	Single	RA	15	2nd and 3rd-degree AV block	SVES, VES, WPW sy 2nd and 3rd-degree AV block	Complete regression	Cortical tubers SENs	Yes	Skin lesions Renal angiolipomas	Autism
9 ^b^	Yes	Clinical		31	Multiple	LV, RV	<5	None	None	Complete regression	Cortical tubers SENs	No	Skin lesions Renal angiolipomas	None
10 ^b^	Yes	Clinical		31	Multiple	LV, IVS	<5	None	None	Regression	Cortical tubers SENs WM migrational abnormalities	No	Skin lesions Renal angiolipomas	None
11	Yes	Genetic	*TSC1*	21	Multiple	LV, IVS	20	VES LVOT obstruction	PSVT, WPW sy Repolarization abnormality LVOT obstruction, MR	Regression	Cortical tubers SENs WM migrational abnormalities	No	None	Moderate
12	No	Genetic	Negative	34	Single	LV	20	None	None	Regression	Normal	No	None	None
13	Yes	Genetic	*TSC2*	24	Multiple	LV, RV, IVS	12	RVOT turbulence	Repolarization abnormality RVOT turbulence	Complete regression	Cortical tubers SEGA	Yes	Skin lesions Optic disc hamartomas Renal angiolipomas	Mild
14	Yes	Genetic	*TSC2*	21	Multiple	LV, RV, IVS	13	Bradycardia SVES, PSVT LVOT obstruction	PSVT, WPW sy LVOT obstruction	Complete regression	SENs SEGA	Yes	Skin lesions Retinal hamartomas and achromic patches	Moderate
15	Yes	Genetic	*TSC2*	26	Multiple	LA, LV	30	None	VES, SVES, PSVT	Complete regression	Cortical tubers SENs	Yes	None	Normal
16	Yes	Clinical		37	Multiple	LV, RV	15	LVOT obstruction	Repolarization abnormality	Regression	Cortical tubers SENs	Yes	Retinal achromic patches	Mild NDI

^a^ Affected twin (the other twin is healthy) ^b^ Both twins affected. Abbreviations: GA, gestational age; IVS, interventricular septum; LA, left atrium; LV, left ventricle; LVOT, left ventricular outflow tract; MR, mitral regurgitation; MRI, magnetic resonance imaging; NDI, neurodevelopmental impairment; PNET, pancreatic neuroendocrine tumor; PSVT, paroxysmal supraventricular tachycardia; RV, right ventricle; RVOT, right ventricular outflow tract; SEGA, subependymal giant cell astrocytoma; SENs, subependymal nodules; SVES, supraventricular extrasystole; Tm, tumor; TR, tricuspid regurgitation; TSC, tuberous sclerosis complex; VES, ventricular extrasystole; WM, white matter; WPW sy, Wolff–Parkinson–White syndrome.

**Table 2:** Frequency of cardiac complications in 16 patients with cardiac tumors before and after birth.

Arrhythmia	Prenatal	Postnatal
Ventricular extrasystole	1	3
Supraventricular extrasystole	1	2
WPW syndrome		3
Paroxysmal supraventricular tachycardia	1	3
Repolarization abnormality		5
Right bundle branch block		1
1st-degree AV block		1
2nd-degree AV block	1	1
3rd-degree AV block	1	1
Hemodynamic disturbances		
Tricuspid regurgitation	1	
Mitral regurgitation		2
LVOT obstruction	3	2
RVOT turbulence	1	1

Abbreviations: AV, atrioventricular; LVOT, left ventricular outflow tract; RVOT, right ventricular outflow tract; WPW syndrome, Wolff–Parkinson–White syndrome.

**Table 3:** Brain MRI findings, neurological outcome, and associated clinical manifestations in neonates diagnosed with TSC (*n* = 14).

Brain Magnetic Resonance Imaging	
Subependymal nodules	11 (79%)
Cortical tubers	10 (71%)
SEGA	4 (29%)
WM migrational abnormalities	3 (21%)
Epilepsy	
Present	10 (71%)
Absent	4 (29%)
Neurodevelopmental Status	
Severe dysfunction	4 (29%)
Moderate dysfunction	3 (21%)
Mild dysfunction	2 (14%)
Normal	5 (36%)
Other Organ Disability	
Skin hypomelanotic macules	11 (79%)
Eyes—retinal hamartomas	3 (21%)
Eyes—retinal achromic patches	4 (29%)
Renal angiolipomas	6 (43%)
Renal multiple cysts	1 (7%)
Pancreatic neuroendocrine tumor	1 (7%)

Data are presented as count (percentage). Abbreviations: SEGA, subependymal giant cell astrocytoma; WM, white matter.

## Data Availability

All the data in relation to this study were stored in the University Hospital Center, Zagreb, and can be presented upon reasonable request to the corresponding author of the published work.
